# Sincerely, Anonymous

**DOI:** 10.1002/tht3.455

**Published:** 2020-04-27

**Authors:** Grace Paterson

**Affiliations:** ^1^ Department of Philosophy University of Vienna Vienna Austria

**Keywords:** anonymity, anonymous speech, identifying information, pseudonyms, speaker intentions, speech acts

## Abstract

This paper provides an account of anonymous speech treated as anonymized speech. It is argued that anonymous speech acts are best defined by reference to intentional acts of blocking a speaker's identification as opposed to the various epistemic effects that imperfectly correlate with these actions. The account is used to examine two important subclasses of anonymized speech: speech using pseudonyms, and speech anonymized in a specifically communicative manner. Several pragmatic and ethical issues with anonymized speech are considered.

## 1 INTRODUCTION

Recently, questions of how to deal with speech acts such as assertion and testimony coming from anonymous and semi‐anonymous sources have come to the fore. Online interactions provide an especially deep pool of situations where we are faced with conflicting claims affixed to anonymous and pseudonymous sources. The aim of this paper is to present a new account of the nature of anonymity. Whereas anonymous speech is standardly defined in terms of the audience's ignorance about the speaker, I will argue that what is of importance is how, in certain cases, the context is manipulated with the aim of concealing in some way a speaker's identity. This view allows that a speaker may be anonymous and yet known to the audience, or that they may be non‐anonymous and yet still unknown to the audience.

There are a number of reasons to prefer this analysis of anonymous speech as anonymized speech to the alternatives defined in terms of epistemic impoverishment. First, it better picks out those cases salient in many debates about anonymous speech while excluding cases that might be considered something of a distraction. Second, it yields a number of useful conceptual resources for more fine grained understanding of specific cases. Finally, it connects naturally to practical questions of responsibility. Of course, this does not mean that there are not interesting questions related to the understanding of epistemically impoverishment—but such obscurity can arise in many ways, only one of which is from having been in some way anonymized. Moreover, once the distinction between epistemically impoverished speech and anonymized speech has been properly drawn, we are equipped to see how the latter can help explain relevant instances of the former. That a speech act has been anonymized in some cases helps explain why one may not be able to identify the speaker; but not being able to identify a speaker is neither necessary nor sufficient for it to be the case that a speech act has been anonymized.

This paper proceeds as follows: in Section 2 I introduce the class of anonymized speech acts and contrast it with the more frequently studied class of epistemically impoverished speech acts. In Section 3 I develop the specifics of my account of anonymized speech, introducing several new theoretical resources. In Section 4, I describe two important subtypes of anonymized speech: pseudonymized speech, and communicatively anonymized speech. In Section 5 I close by briefly considering how this account is better equipped to deal with pragmatic and ethical issues related to anonymous speech than epistemic impoverishment centered approaches.

## 2 ANONYMIZED SPEECH ACTS

The account of anonymity I propose takes as its central element and starting place the act of anonymizing a speech act. Thus on my view *anonymous speech* is more correctly termed *anonymized speech*. A more precise definition of an anonymized speech act as follows:

***Anonymized speech act*:** A speech act is *anonymized* when the audience is intentionally blocked from identifying the speaker.

This contrasts with accounts of anonymity which focus not on any intentional act but rather on the knowledge and beliefs of the participants, especially the audience. The specifics vary across accounts, but the general idea on such views is that anonymity is a matter of the audience not identifying the speaker or not being able to identify the speaker. Thus, anonymous speech is speech that is, in some way, *epistemically impoverished*.

More specifically such accounts usually define anonymity in terms of the following conditions of epistemic impoverishment, taken in various combinations.^1^ (Note that the modal variants of these conditions help to exclude cases in which the epistemic failure of the audience is due to their own negligence or inattention rather than a feature of the actual speech act or situation.)

***Conditions of epistemic impoverishment*:**

1. The audience has not identified (or cannot identify) the speaker.
2. The audience believes that they have not identified (or cannot identify) the speaker.
3. The speaker believes that the audience has not identified (or cannot identify) the speaker.

Unsurprisingly, many speech acts happen to be both anonymized and instances of some form of epistemic impoverishment. For instance, consider a prank call where the caller makes a point of not identifying themselves. Now this is, in the impoverishment sense, an anonymous call: the audience cannot identify the speaker and, indeed, both speaker and audience believe that the audience is ignorant in this way. Moreover, it is also an anonymized call, since the caller chose to conceal their identity. Similarly, unsigned letters or books published without an author's name (e.g., the first edition of Frankenstein) are instances of both anonymized speech and epistemically impoverished speech.

That said, there are also many cases of epistemically impoverished speech that are not anonymized. Here is an example: two people meet in a club and try to introduce themselves. Unfortunately, the lights are too dim for them to see one another properly and the music is too loud for them to make out each other's names despite shouting. Now since both participants (correctly) realize that the other has no means of identifying them, this is clearly a case of speech in a context of epistemic impoverishment. These speech acts have not, however, been anonymized—nobody is trying to hide their identity. In fact, in this scenario both participants are obviously trying to make themselves known to the other. So this is an example of epistemic impoverishment, but it is not a case of anonymized speech.

Another kind of speech that is epistemically impoverished but not anonymized is that where the audience is prevented from identifying the speaker on account of something accidental. We see this sometimes with texts where information about the author has been lost but where the text was never intended to be anonymous (many ancient texts fall in this category). Just as a book may be published anonymously and the identity of the author revealed at a later date (we now know Mary Shelley wrote Frankenstein), it can also be the case that the text is published openly but the identifying information about the author may be lost over time. These are texts received through a haze of epistemic impoverishment, but they were never anonymized. Grouping these cases together with speech acts that have been anonymized strikes me as misleading. Surely not knowing who an author is because of information loss can have very different effects on how we interpret that text from those that come from knowing that a text was anonymized (for instance, it is only the latter that raises questions about the political climate under which the text was authored). And, in fact, I think there is something intuitive about this distinction: while texts with unknown authorship are sometimes attributed to “anonymous” they are also sometimes attributed to “author unknown.” This terminology is suggestive of the difference between anonymized speech and accidental epistemic impoverishment, even if its usage is not systematic in everyday practice.

On the flip side, anonymized speech may generate little (if any) impoverishment. Indeed the identity of the speaker of an anonymized speech act may be widely known (or suspected). For instance, some authors who write anonymously (or pseudonymously) reveal their identity within that very same text. In such cases, anonymity is driven by expressive rather than properly epistemic motives. As another example, some anonymizing of speech functions more like an invitation not to pry or make use of one's information about the speaker. In these cases the conditions of epistemic impoverishment may not be met.

Anonymized speech acts and epistemically impoverished speech acts are, therefore, overlapping but distinct classes. On my view, anonymized speech is the more appropriate target class for most philosophical applications, especially those in ethics and philosophy of language. This because it is the class to which the most relevant questions apply. When a speech act is epistemically impoverished we may ask *how* such a circumstance came to be and *what effects* this has; but when a speech act is anonymized, we may also ask *why?*, *by whom?*, and *to what end?*. These are all questions pervasive in debates about anonymous speech.^2^

## 3 IDENTIFICATION AND BLOCKING

Above, I defined an anonymized speech act as a speech act where the audience is intentionally blocked from identifying the speaker. In this section, I will explain more precisely what is meant by *identification* and *blocking*.

Let us begin with identification. I take identifying the agent of an action to be a matter of correctly associating the agent in question with other facts about them:

*Identification*: One has identified an agent of a particular action A when either (a) one knows that specific facts hold of the agent of A which are not directly related to A, or (b) one can be reasonably expected to come to know such facts in the future.^3^

On this view, identification is a matter of connecting the agent to facts unrelated to the action itself. In order for us to draw such connections, we must make use of information (including qualitative information) that accompanies the action in question. As a rule, the more we interact with someone and can identify them in these interactions, the more information related to them we will acquire and the easier it will be for us to identify them in the future.

This “identifying information”^4^ can take many forms. We are, for instance, able sometimes to identify a person who has spoken to us based on visual or auditory information—how they look or sound. Alternately we may have more explicit identifying information such the speaker's postal address. An address may be used to help us identify the sender as the author of other letters coming from the same place but is of little use in identifying them at the movie theater or on the street where we must depend instead on sensory information.

It should be fairly evident that almost any piece of information can be used to identify the speaker in the right sort of circumstance. An accent, mannerism, or turn of phrase may be distinctive and unusual enough within a particular (and perhaps itself idiosyncratic) social setting to make someone identifiable, even if in most other settings these same things would not be distinctive at all. Learning biographical facts about someone can help you identify them in situations where those details are mentioned again or come up in some other way. Then again, they may not. The fact that I like to wear fancy hats may help you pick me out in a crowd at a subway station, but not in a hat store, or at a hat fancier's convention, or at a subway station on the way to a hat fancier's convention. Such complications notwithstanding, the more information available about a speaker, the more identifiable they are likely to be.

When a speaker is unidentifiable, the audience only knows of the speaker *qua* speaker of that act what they can learn through that very speech act. Such a speech act is, in this way, isolated for the hearer from everything else the speaker says or does. Unidentifiability of an agent is typically a result of there being one or more *blocks* to identification, understood as follows:

*Block to identification*: a block to identification is anything that makes the identification of the agent of a particular action more difficult.

Anything or anyone that itself constitutes a block to identification is said to be *blocking*.^5^ Similarly, anything or anyone that creates a block to an agent's identification is *blocking* its identification. Identification of an agent is *blocked* when either someone is engaged in blocking or there are other sorts of blocks present. In general, the more and better the blocks to identification, the harder the agent will be to identify. Moreover, blocking is not used here as a success term; an agent may be identifiable to some degree (perhaps even a high degree) in spite of her identification having been blocked. Similarly, blocks may be partial in the sense that they may allow the speaker to be concealed or identifiable in highly circumscribed ways. These points will play an important role in what follows. Partiality allows for pseudonymous speech acts, while gradability means that one can have communicatively rich forms of anonymized speech with deceptively weak blocks.^6^

## 4 VARIETIES OF ANONYMIZED SPEECH

With a conception of anonymized speech in place, let us turn now to two subtypes of anonymized speech: pseudonymized speech and communicatively anonymized speech. Notice that these are not mutually exclusive: speech acts can be, and often are, both pseudonymous and communicative.

Let us begin with pseudonymized speech. The idea here is that the kind of information an audience member has about a speaker can constrain how and when they are able to identify that speaker. This is the sense in which an individual may be identifiable under one “guise” and not another. For instance, Lois Lane does not connect the actions she witnesses performed by her colleague in glasses with those performed by the caped superhero. It is not that Superman is entirely non‐identifiable to Lois. Rather, the point is that when wearing his costume he is identifiable to her only *as* Superman and not *as* Clark Kent. Many pseudonyms are employed to constrain identifiability in precisely this manner. They allow for the speaker to be circumstantially identifiable: all and only their actions performed in particular ways (i.e., those marked by the pseudonym) can be associated together. More precisely, pseudonymized speech can be defined as follows:

*Pseudonymized speech act*: a partially or circumstantially anonymized speech act.

The thought is that I may have online interactions with someone using a pseudonym and not realize that this pseudonym belongs to a good off‐line friend. I may be familiar with Banksy as an artist, and have no idea that I have met him in person. A pseudonym facilitates identification within a constrained setting while simultaneously presenting a block to identification otherwise.

Another related subtype of anonymized speech is what I will call *communicatively anonymized speech*. Communicatively anonymizing a speech act involves making a point of blocking identification of the speaker in a manner that is meant to be noticed. For example, disguising your voice over the phone, wearing a mask, not signing your name, or failing to include a return address with a letter can all be means of quite openly blocking identification.

The point applies to the use of pseudonyms as well: speech acts can be pseudonymized either communicatively or non‐communicatively. Edward Gorey, in using playful anagrams of his own name (e.g., “Ogdred Weary”), was pseudonymizing his work communicatively; Mary Ann Evans, by using the entirely natural sounding pseudonym “George Eliot” so as to conceal her gender was also pseudonymizing her work, but she was not doing so communicatively.

What sets communicative anonymizing (and pseudonymizing) apart from its non‐communicative siblings is that the audience is being made aware of the fact that there is some information they lack, and are intended to lack. In short, the fact that a speech act is anonymized is marked.

There are a number of reasons it can matter whether a speech act that has been anonymized has, in fact, been *communicatively* anonymized. First, it can give the audience an opportunity to respond appropriately to the anonymity by, for instance, adjusting their credences or adopting a more skeptical stance. The speaker may not be accountable in an epistemically ideal way, but at least this is made transparent to the audience.^7^

Second, it can raise to salience aspects of the communicative context that were relevant to the decision to anonymize such as that the speaker feels vulnerable or considers her discursive context hostile. Thus, the whistleblower who speaks anonymously may be making a point about the risks they are taking in addition to attempting to protect themselves from retaliation.

Third, the blocks themselves may be of interest. Indeed, I suspect that the expressive ends served by the pseudonyms of “Cato,” “Caesar,” and “Brutus” in 18th century American political discourse^8^ would have been more seriously undermined by the audience not recognizing the relevant classical references than by the author being exposed.^9^

Let us define communicative anonymizing more precisely:

***Communicatively anonymized speech act*:** A speech act is communicatively anonymized when:

1. The audience is intentionally blocked from identifying the speaker by way of information available to any reasonable, attentive, and linguistically competent audience member in virtue only of being in the audience.
2. The intention to block the speaker's identification is intended to be recognized as such by the audience.
3. All of this is out in the open, such that all conversational participants can reasonably expect all other conversational participants to know (1), (2), and (3).

The first condition here is, in the main, the definition of anonymizing which we have already seen; however something has been added. This is that the audience is blocked from identifying the speaker specifically by way of information available to any reasonable, attentive, and linguistically competent audience member in virtue only of being in the audience. What is important here is that this excludes from consideration certain methods of identification which one might characterize as lucky, circumstantial, or forensic.

The thought here is that the information that must be blocked is only that which requires no unusual expertise, skill, or background knowledge to recognize and make use of. One kind of identifying information that need not be blocked for communicatively anonymizing a speech act is that which is about the speech act but which does not come through the speech act itself. Suppose, for instance, that there is a piece of graffiti art with no signature. In general, when people see this piece of art they will have no way of identifying its creator because on its own it lacks identifying information. Now if someone confesses to a reporter that they were the one who created that piece of graffiti, then they will be identifiable to that reporter as the graffiti artist. But they are identifiable in virtue of information that is external to the art itself.

In fact, even if the reporter were to widely publicize the identity of the graffiti artist, thereby creating a context in which their identity was widely (or even universally) known, it is possible for the art retain its status as communicatively anonymized. And indeed, in most cases I think it would, as an audience member would have to use information external to the piece to connect the dots in the manner required to identify the creator. They would, moreover, be reasonably expected to recognize the blocks inherent in the piece itself. Thus, they could recognize the anonymizing intent while also being aware of who was behind it. That said, it is also possible for such an interview to result in a cultural context which essentially results in its being *de‐anonymized*.^10^ For this to occur, the identity of the creator would have to become common background knowledge. Such widespread knowledge would have to obscure the blocks such that it would not longer be reasonable to expect an audience member to pick up on them. Once this occurred, it would actually surprise most people to learn that the piece had originally been in any way anonymized. We can see this kind of de‐anonymizing with such works as Mary Shelley's Frankenstein: its authorship is now so widely known that many people are unaware that the book was originally anonymized. Moreover, most new editions and adaptations are attributed to her, removing any obvious trace of the original blocks.

Another kind of identifying information which need not be blocked is that which is not actually recognizable or usable by all participants. So, for instance, some things might be too subtle for it to be expected that all conversational participants will grasp them. As a rule, it is much harder to fool Sherlock Holmes than it is to fool Dr Watson—not because Holmes has more information available to him but rather because he notices more of the available information than Watson does. It is only what us Watsons of the world are likely to notice that must be blocked when communicatively anonymizing speech.

The second condition in this definition says that the intention that the audience not be able to identify the speaker is intended to be recognized as such. This is meant to exclude cases of deceit such as impersonation in which the audience is intended to falsely believe that they can identify the speaker as well as cases in which they are intended to falsely believe that their inability to identify the speaker is by accident rather than design.

Finally, the third condition ensures that something like common knowledge obtains, such that all conversational participants are aware that these conditions obtain, and can reasonably expect that all other participants are also aware.

We can see from this definition that what matters to communicative anonymity is not that a speaker's blocking of the relevantly identifying information is especially effective but rather that the blocks have been made obvious to the audience. In fact, it is striking that in certain circumstances, a speaker may need do no more than announce that they wish to be anonymous. An especially friendly and cooperative audience member may go along with this, or even try to help the speaker. They may glance away when they see a mask slipping, ignore that which they realize could be used to identify the speaker in the future, refrain from asking for obviously identifying information, and, if they do to happen recognize someone who has in this way anonymized their speech, they may even pretend not to have done so.

## 5 CONCLUDING REMARKS

An anonymized speech act is subject to moral and rational appraisal to determine whether the anonymizing devices are warranted. A speaker who actively blocks her own identification is answerable for the blocks and their effects in a way that a speaker who is subject to environmental noise or historical accident is not. Thus, for instance, someone who anonymizes her own threat is, by way of her anonymizing it, attempting to thwart efforts to protect her victim. Similarly, someone who anonymizes her assertion is actively making it more difficult for her audience to check her credentials or ask follow‐up questions.^11^ In both these cases, the speaker is responsible for the predicament the audience is in. How this is to be evaluated will depend to some extent on their reasons for placing the audience in such a predicament. Adding further complexity, when the speech act is communicatively anonymized, this may alter the speech act's uptake and perlocutionary effects because the speaker is raising the salience of the anonymizing devices, flagging it as in some way important.

There is much to be said here—the specific consequences and implications of anonymizing speech will depend on a variety of factors. A whistleblower leaking sensitive information or filing a complaint and who fears retaliation has, we might think, a good reason to anonymize her assertion even if this makes it harder for the information she presents to be assessed. Indeed, the environment may be so toxic as to have a coercive effect on her—essentially forcing her to anonymize her speech. These facts will change how we assess the speech act on many levels. Coerced anonymity does not indicate uncooperative behavior in the way freely chosen anonymity does.

On the other hand if a terrorist anonymizes a threat because they fear “retaliation,” that reason is hardly exculpatory. It is certainly rational for them to do this—if communicatively done, it might amplify the intended perlocutionary effect of generating fear—but the vicious nature of the speech act itself precludes such excuses from being persuasive.

A full study of the rich dynamics of these and other cases is beyond the scope of this paper. But notice that, in order to address these issues, an account built around epistemic impoverishment must be supplemented with a discussion of how and why speakers may choose to speak in, or create for themselves, epistemically impoverished contexts.^12^ Such maneuvers are symptomatic of the fact that analyzing anonymity by beginning with certain epistemic effects that it often generates puts the explanatory cart before the horse. Given this, I think we should take as our starting place the intentional acts that characterize anonymized speech rather than the epistemic fallout of these acts.

## ENDNOTES

1 Some examples: Matheson (2004) and Marx (1999) employ notions of anonymity that are roughly equivalent to (1). A heavy focus on (1) (with occasional appeals to (2) and (3)) may also be seen in discussions of anonymity in legal scholarship, for instance Levmore (1996). Scott (1998) has an account of subjective anonymity combining (2) and (3), with Rains and Scott (2007) developing (2) in more depth. Goldberg (2013) combines aspects of (1) and (3), with an additional provision that the identity of the speaker is “hidden” from the audience (see fn 6).

2 For instance, In his monograph, Barendt (2016) aptly identifies “the central problem of anonymity” as “how to reconcile the interest of a writer in protecting his identity from public disclosure with the need for responsibility and accountability to injured readers” (2).

3 This has some commonality with the account of *knowing who* in Boër and Lycan (1986), and accords with Goldberg's gloss of knowing who as having “identificatory knowledge of the speaker or writer that would enable to reidentification of S *as* the speaker or writer of the message” (Goldberg, 2013, p. 136). Cf. also Wallace (1999) where anonymity is defined as “noncoordinatability of traits in a given respect”.

4 See Marx (1999) for a taxonomy of some prominent kinds of “identity knowledge.”

5 Note that the notion of blocking given here is different from the notion of blocking that appears in Langton (2018). Langton is concerned with blocking in the sense of resisting, rather than accommodating, a speech act's presupposition. For instance, the speech act “even George could win” brings in a presupposition disparaging George's competence. But this presupposition can be *blocked* by replying “whaddya mean, *even*” (Langton, 2018, p. 153).

6 Being blocked is comparable to being “hidden” in Goldberg (2013) with a few significant caveats. First although Goldberg does not tell us if “hidden” is a success term, he takes an anonymous assertion to be one which is epistemically impoverished *because* the speaker's identity is hidden from the audience. Consequently, his account specifically targets instances of hiding which do in fact succeed at creating contexts of impoverishment relative to the audience. Second, although he seems most concerned with the intentional cases, Goldberg does not distinguish between being intentionally and unintentionally hidden. To explain “hiding,” he tells us that it rules out cases such as when an author puts her name on a book but none of her readers look at the cover (pp. 136–137). However, this would be compatible with either an intentional or non‐intentional reading. These points mean that his view excludes many interesting instances anonymized speech, especially communicatively anonymized speech. For instance, while his relativization of anonymity to a particular audience does accommodate a certain subset of pseudonymized speech, it still excludes the sorts of artistic uses where the success of the blocking is largely beside the point.

7 This is consistent with certain norms of transparency in journalism. For instance, the NPR ethics handbook specifies that when reporting on anonymous sources, there should be “No disguises: We may withhold a source's name (…) We may refer to the person without using a last name (…) But we don't use pseudonyms to replace their real name” (NPR, 2012).

8 For discussion of these pseudonymized debates, see Shalev (2003).

9 Such pseudonymous speech acts feature prominently in debates about the interplay between anonymity and a right to free speech. For instance, In their review of Barendt (2016), Heinze and Hannant (2018) argue that there is a persuasive case to be made for a right to anonymous speech insofar as “choosing identities like “Concerned Citizen” or “Anon” (…) can frame and pervade the entirety of a message” (Heinze & Hannant, 2018, p. 367). It is of interest, I think, that this point applies specifically to communicatively anonymized/pseudonymized speech, not to anonymized speech in general. Moreover, since the information communicated by communicatively anonymizing speech need not be undermined by speaker exposure, a right to communicatively anonymize one's speech also needn't entail a right to all out obscurity.

10 My thanks to an anonymized referee for raising this point.

11 Notice that this is compatible with—but stronger than—the claim in Goldberg (2013) that the audience has “generic grounds for skepticism regarding the truth of assertions presented anonymously” (p. 147). On my view, that she is placing additional epistemic burdens on her audience to raise skeptical worries even if they aren't successful. At the same time, the nature and strength of the blocks is also relevant. So, for instance, weak and communicative blocking might suggest expressive rather than evasive motives.

12 For example, Levmore (1996) places much significance on a communicator's choice to speak either anonymously or non‐anonymously. Levmore also discusses the role of speaker's self‐identification (including false identification) in signaling certain things to an audience.
